# A Rare Case and Presentation of Traumatic Penetrating Aortic Arch Injury: A Case Report and Literature Review

**DOI:** 10.7759/cureus.31069

**Published:** 2022-11-03

**Authors:** Mohammed N AlAli, Mohamed S Essa, Muath Alasheikh, Muath Alrashed, Abdullah M Albdah, Arief Arrowaili

**Affiliations:** 1 General Surgery, Prince Mohammed bin Abdulaziz Hospital, Riyadh, SAU; 2 Trauma Surgery, Prince Mohammed bin Abdulaziz Hospital, Riyadh, SAU; 3 General Surgery, College of Medicine, Al-Imam Muhammad Ibn Saud Islamic University, Riyadh, SAU

**Keywords:** surviving trauma, supraclavicular trauma, median thoracotomy, chest injury, aortic arch, penetrating trauma

## Abstract

A penetrating injury to the thoracic aorta is an extremely rare, life-threatening condition, with a high overall mortality rate. The incidence of a penetrating injury to the aortic arch is unknown because the majority of patients die before receiving adequate treatment due to excessive bleeding. Through a literature review, 23 cases of favorable outcomes were found. We report the first case from the Arab Gulf states. We present the extremely rare case of a 23-year-old male who presented to the emergency department with stable hemodynamics after being stabbed in the left supraclavicular region. The investigation revealed that he suffered from aortic arch transection and contrast extravasation. The patient was rushed to the operating room, where a primary repair was performed through a median sternotomy approach. The patient was discharged on the 14th postoperative day without complications. Penetrating chest trauma (aortic arch injury) is uncommon, and it is typically fatal at the scene or time of injury, even in patients who arrive at the emergency department alive or while undergoing surgery. CT aortography should be performed on patients with normal vital signs but abnormal clinical findings suggestive of a vascular injury. For injuries of types II to IV without concomitant injuries, immediate surgical repair is recommended. Aortic arch penetrating injuries continue to be extremely lethal. Emergency surgical repair remains the standard of care and is associated with high morbidity and mortality rates. However, managing such uncommon injuries remains a formidable challenge. We encourage additional studies.

## Introduction

Penetrating thoracic trauma (PTT) occurs at varying rates across the globe, accounting for 9% of all trauma-related deaths in the United States but only 4% in Europe [1-3]. A penetrating injury to the thoracic aorta is an extremely rare entity and a life-threatening condition associated with a high risk of mortality reaching up to more than 90% [4]. The incidence of a penetrating injury to the aortic arch is unknown because the majority of patients die due to severe bleeding (estimated to be between 4% and 25%) before receiving adequate treatment [4-6]. In Saudi Arabia, the incidence of a thoracic stabbing mechanism affecting the major vessels is 3.4% (based on a single pediatric age group study; mean = 15.5±3.6 years) [7].

Clinical presentations of such injuries are either external or internal bleeding, bruit, pulse deficit distally, neurological deficit, and hemorrhagic shock [4]. An aortic injury is usually diagnosed by an abnormal chest X-ray, extended focused assessment with sonography for trauma (E-FAST), or computed tomography (CT), followed by more definitive investigations. Therefore, rapid detection and decisive intervention are paramount. However, the majority of such aortic trauma patients are treated in hospitals where cardiac and vascular surgeons are unfamiliar with these complicated aortic injuries [8-10].

To the best of our knowledge, penetrating aortic arch injuries are rare, and such presentations and outcomes are even rarer worldwide. Therefore, we are the first to report a case from Saudi Arabia and the Arab Gulf states.

## Case presentation

**Figure 1:**
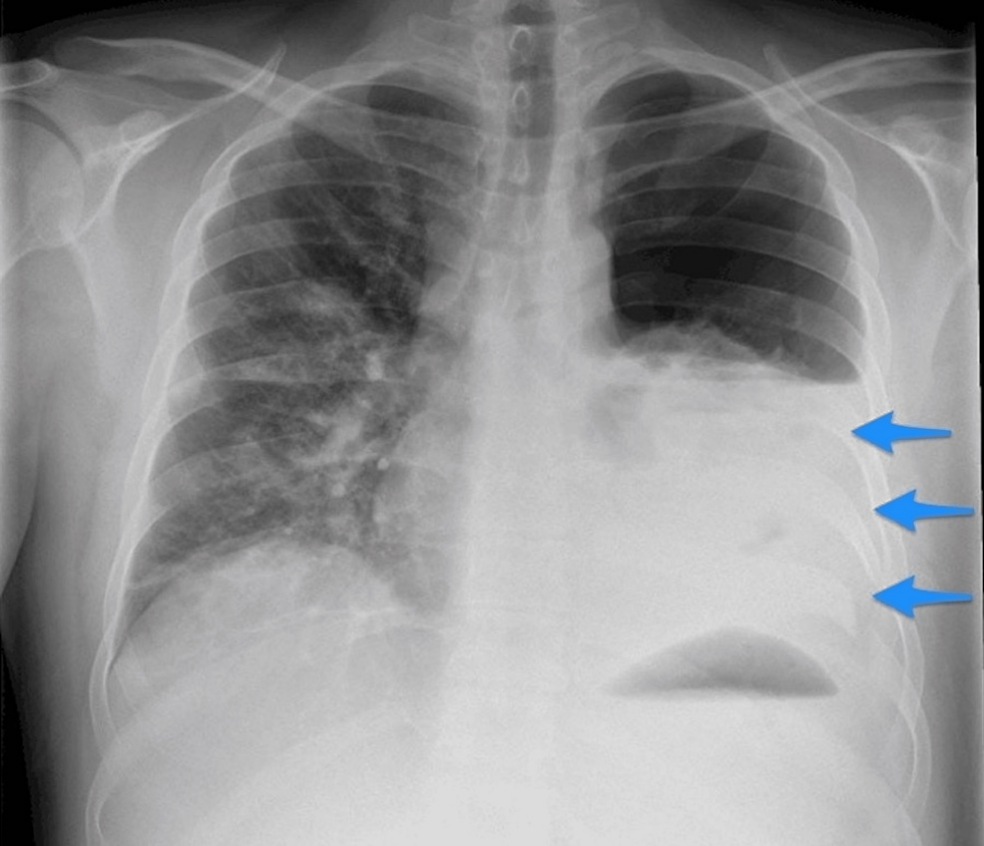
Left-sided hemopneumothorax (arrows).

A 23-year-old male presented to the emergency department after being stabbed in the left supraclavicular region (the knife was already removed). The Advanced Trauma Life Support (ATLS) protocol was followed during the patient's management. The patient presented with a heart rate of 95 beats per minute, blood pressure of 110/70 mmHg, a blood oxygen saturation of 100% on room air, and reduced air entry on the left side of the chest. The chest X-ray revealed hemopneumothorax on the left side (Figure 1). A chest tube was inserted, and 300 milliliters (mL) of blood were extracted. There were no indications of pericardial or peritoneal fluid on E-FAST. Laboratory workups were performed, including hemoglobin (10 g/L; reference range: 13.2-16.6 g/L), hematocrit (30%; reference range: 41%-50%), leukocytes (21.6 x 10^9^/L; reference range: 4.5-11.0 × 10^9^/L), venous blood gas (pH 7.37; reference range: 7.35-7.45), base deficit (-8 mEq/L; reference range: -4 to +4 mEq/L), HCO_3_ (15.8 mEq/L; reference range: 20-24 mEq/L), and international normalized ratio (INR) (1.18; reference range: ≤1.1), while the others were unremarkable.

**Figure 2:**
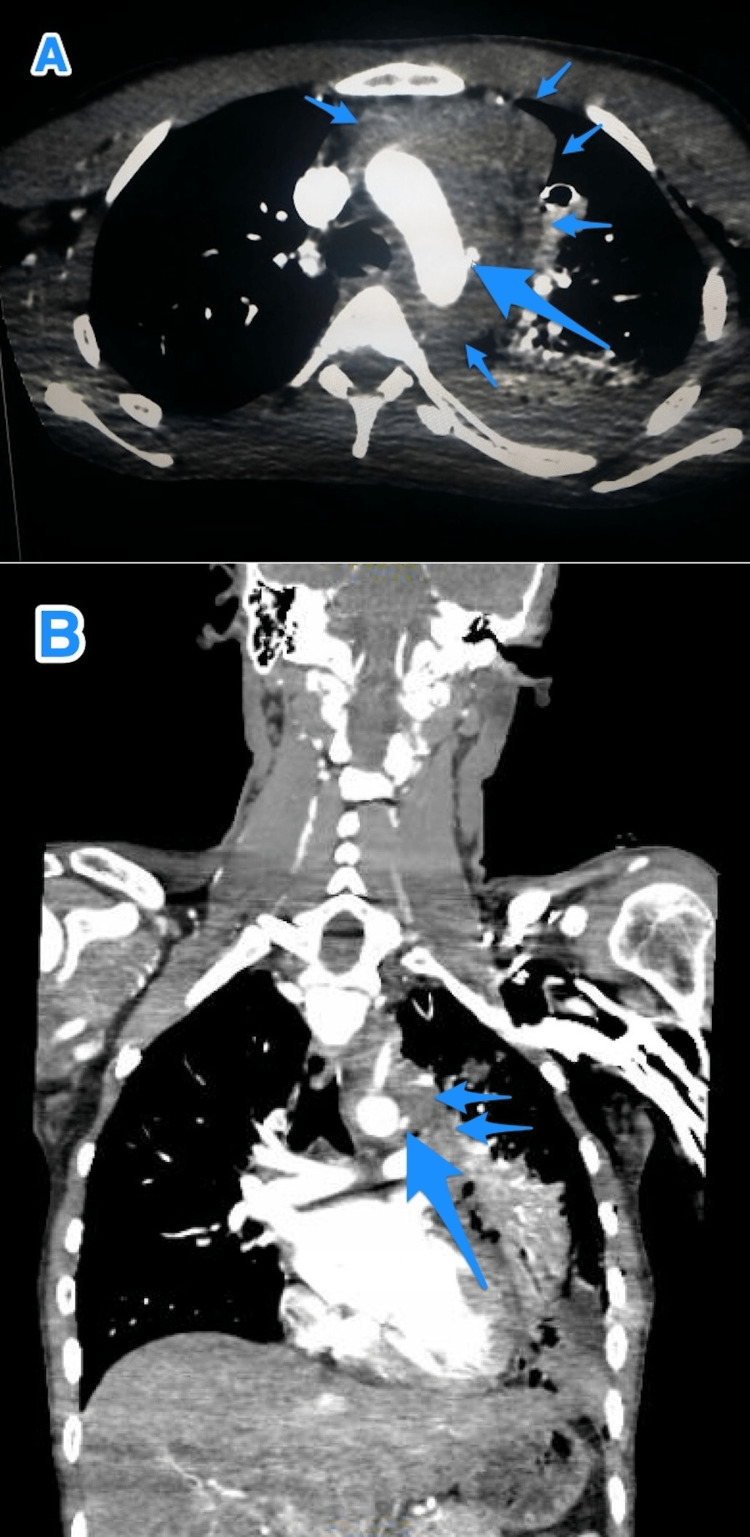
(A and B ) A chest CT angiography shows an aortic arch injury with extravasation of contrast (large arrow) about 1.5 cm from the left subclavian artery (zone 3) and surrounded by a large hematoma (small arrows).

**Figure 3:**
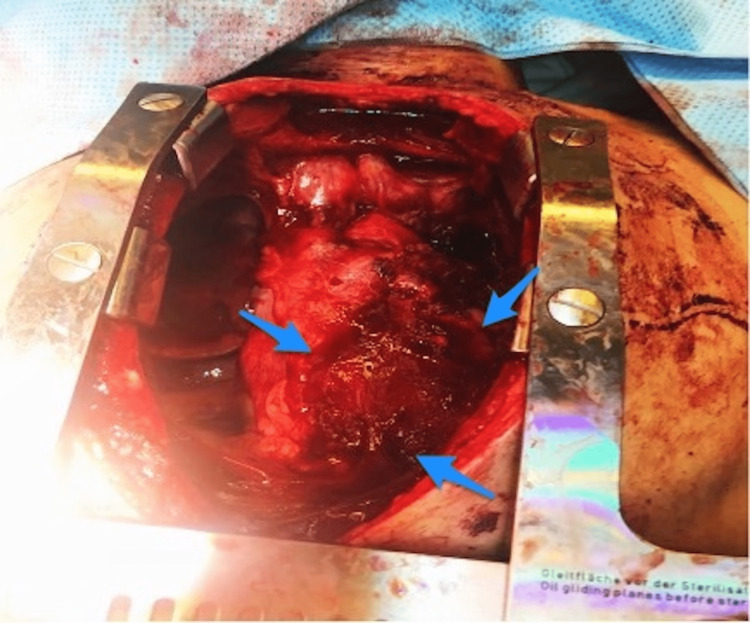
Intraoperative finding of large mediastinal hematoma (arrows).

A CT angiography was performed, which revealed that there was a transection at the aortic arch distal to the left subclavian artery (zone 3), in conjunction with contrast extravasation, leading to the formation of a moderate-to-severe left hemothorax and a large mediastinal hematoma (Figure 2). The patient was rushed to the operating room, where a median sternotomy was performed, and a large mediastinal hematoma was discovered (Figure 3). A dissection of the aorta was performed to allow for proximal and distal control of the aortic arch. However, an aortic arch injury of about 0.5 cm in size at the superolateral aspect was discovered, which was actively bleeding. A single polypropylene 4-0 stitch was applied to control the bleeding and repair the defect. To achieve hemostasis, Oxicel Absorbable Hemostat was applied at the site of the repair. The total blood loss was estimated to be about 800 mL. An additional chest tube was inserted before closing the median sternotomy incision. The patient was transferred to the intensive care unit (ICU), while he was intubated and extubated on the second postoperative day, and the chest tube was removed on the 10th postoperative day.

**Figure 4:**
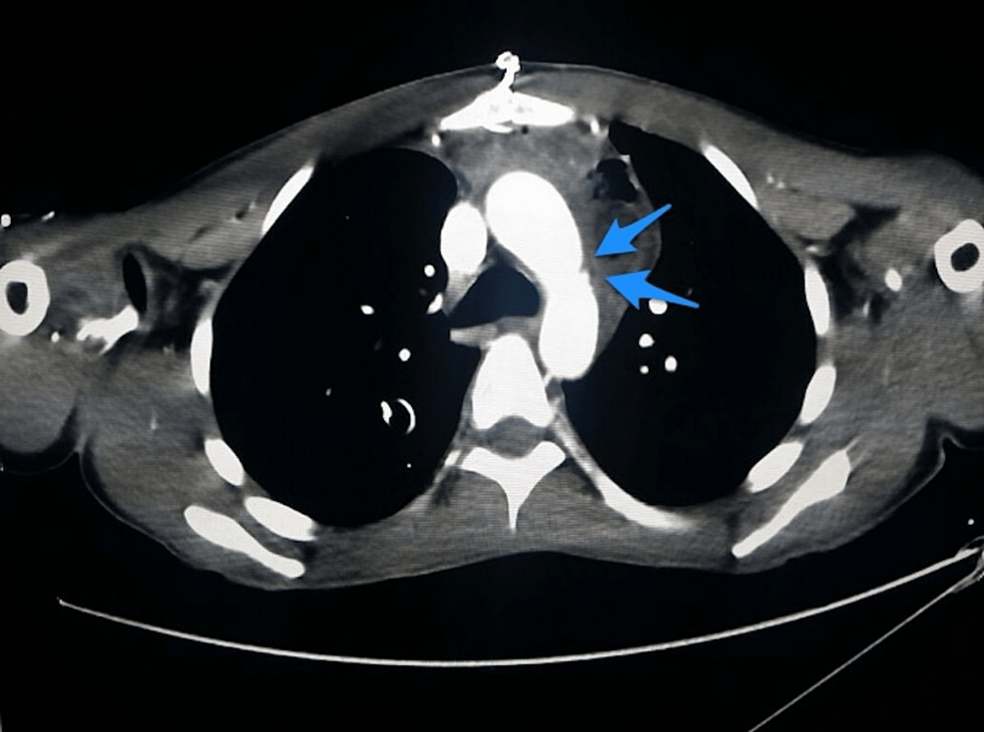
Follow-up axial CT angiography demonstrates no contrast extravasation with irregularity in the wall (arrows).

During the hospital stay, apart from the intraoperative transfusion of one unit of packed red blood cells and four units of fresh frozen plasma, the patient did not require any blood or blood products to be transfused, and follow-up was unremarkable with serial chest X-rays and CT angiography. The latter revealed an irregularity of the lateral aspect of the aortic arch corresponding to the previous site of aortic injury with no contrast extravasation (Figure 4). On the 14th postoperative day, the patient was discharged without complications. The patient was seen for regular outpatient clinic follow-ups (two, six, and 16 weeks after discharge), and he was asymptomatic.

## Discussion

PTT is less prevalent than blunt chest trauma and is associated with a higher mortality rate (mostly from gunshots and stabbings) [1,3]. However, there are no data reflecting the incidence in the Arabian Gulf states or even in the Middle East. In countries or regions engaged in warfare, up to 95% of military deaths may result from a penetrating mechanism [11]. In contrast to our case and the other cases (about 23 cases) [12-33] found through a literature review conducted using PubMed and Google Scholar of people who survived penetrating aortic arch injuries, these injuries are usually fatal at the scene or time of injury, even in people who make it to the emergency department or operating room alive [3].

When comparing a penetrating thoracic injury to a blunt injury, in addition to the clinical manifestation, the entrance and exit wounds are essential factors in figuring out the course and consequences of the injury. When patients are hemodynamically stable, a vascular injury may be masked by other associated injuries to vital structures in the chest. Patients may present with hemorrhage, cardiac tamponade, or systemic air embolism, which can be detected early on anteroposterior chest radiography, the most important initial screening tool. It can show multiple signs such as widening of the superior mediastinum >8 cm and/or 25% of the width of the thorax (most common), loss of aortic knob, the obvious double contour of the aorta/abnormalities of the transverse aortic arch (sensitive finding), obliteration of the aortopulmonary window on lateral chest radiography (sensitive finding), massive hemothorax, and right deviation of the nasogastric, endotracheal, or esophageal tubes [8]. Normal vital signs with abnormal findings suggestive of a vascular injury should undergo CT aortography, the gold standard test, to confirm the diagnosis and facilitate operative planning since aortic injury grading is classified based on CT findings [25,34]. Signs on CT include abnormal aortic contour, sudden change in aortic caliber (pseudocoarctation), contained rupture, traumatic pseudoaneurysm, intimal flap, and intraluminal mural thrombus [25]. Alternatively, magnetic resonance imaging (MRI) of the thorax, conventional angiography, intravascular ultrasound (IVUS), or transesophageal echocardiogram (TOE) can be used as needed [25,34].

For type II, III, and IV injuries without concomitant injuries requiring emergency intervention such as laparotomy, craniotomy, or pelvic stabilization, immediate surgical repair is advised [25]. In the literature, there are cases of a contained penetrating thoracic aortic arch injury that were successfully treated with a non-operative approach, which may be a line of management in certain patient groups in the era of the magnificent development of higher resolution CT scans [25]. If surgical treatment for a contained aortic injury is delayed, patients should be aggressively treated with short-acting beta-blockers to reduce the ongoing wall stress sustained by the aorta and the incidence of aortic rupture. The indications for urgent surgical intervention in penetrating aortic injury are initial loss of 1,500 mL of blood from the chest tube, continuing hemorrhage of >200 ml/hour from the chest tube, hemopericardium, cardiac tamponade, exsanguinating hemorrhage presenting from the supraclavicular wound, evidence of acute thoracic great vessel injury on imaging or radiographic, or other imaging evidence of chronic thoracic great vessel injury complications [25]. Therefore, since our patient was hemodynamically stable and CT angiography showed aortic arch transection with contrast extravasation, he underwent median sternotomy with the aortic arch repair without the use of cardiopulmonary bypass (CPB) or deep hypothermic circulatory arrest (DHCA) [2]. The CPB or DHCA provides ideal exposure to the aortic arch in a bloodless field, allowing the surgeon to examine the entire aortic arch from inside the lumen and to protect the brain when great vessel injury or uncertainty about the degree of the damage coexists [23]. Furthermore, the choice of incision depends on the location of injury with further extension if needed [23]. Proximal and distal control of the aorta should be initiated prior to the primary repair of small anterior or lateral lacerations using continuous 4/0 polypropylene sutures. Larger or more complex injuries may require repair and reconstruction using interposition grafts and CPB [25].

In the last few years, the endovascular approach has been evaluated as an alternative to the conventional surgical approach [35]. Patients with stable mediastinal connective tissue and relatively low and stable blood pressure are the most suitable candidates for endovascular therapy. As a result, injuries to the descending thoracic aorta are more amenable to endovascular options due to their location and stability, whereas proximal injuries, such as those to the aortic arch, are rarely candidates because these patients typically require more urgent surgical intervention [35]. In addition, special considerations are required for the endovascular approach: an adequate "seal or landing zone" for the stent graft and lifelong imaging monitoring (30 days, 6 months, and 12 months after repair, then annually if there are no complications) [35,36].

## Conclusions

Penetrating aortic injuries, including penetrating aortic arch injuries, remain highly lethal. Generally, emergency surgical repair is still the standard of care even in the presence of the new era of endovascular treatment and is associated with high morbidity and mortality. However, dealing with such rare injuries remains highly challenging, especially if they are associated with unusual presentations. Further reports and larger national studies are encouraged to determine the real prevalence and presentation of patients who survived such a fatal injury as well as the feasibility of adopting conservative management.
